# Supplementary material for: Effect of Purslane (Portulaca oleracea L.) on Intestinal Morphology, Digestion Activity and Microbiome of Chinese Pond Turtle (Mauremys reevesii) during Aeromonas hydrophila Infection
Source: Int J Mol Sci. 2023 Jun 17;24(12):10260. doi: 10.3390/ijms241210260 (PMC10298896; doi:10.3390/ijms241210260)
Supplement: Supplementary file 1 [file ijms-24-10260-s001.zip › ijms-2421065-supplementary.pdf]

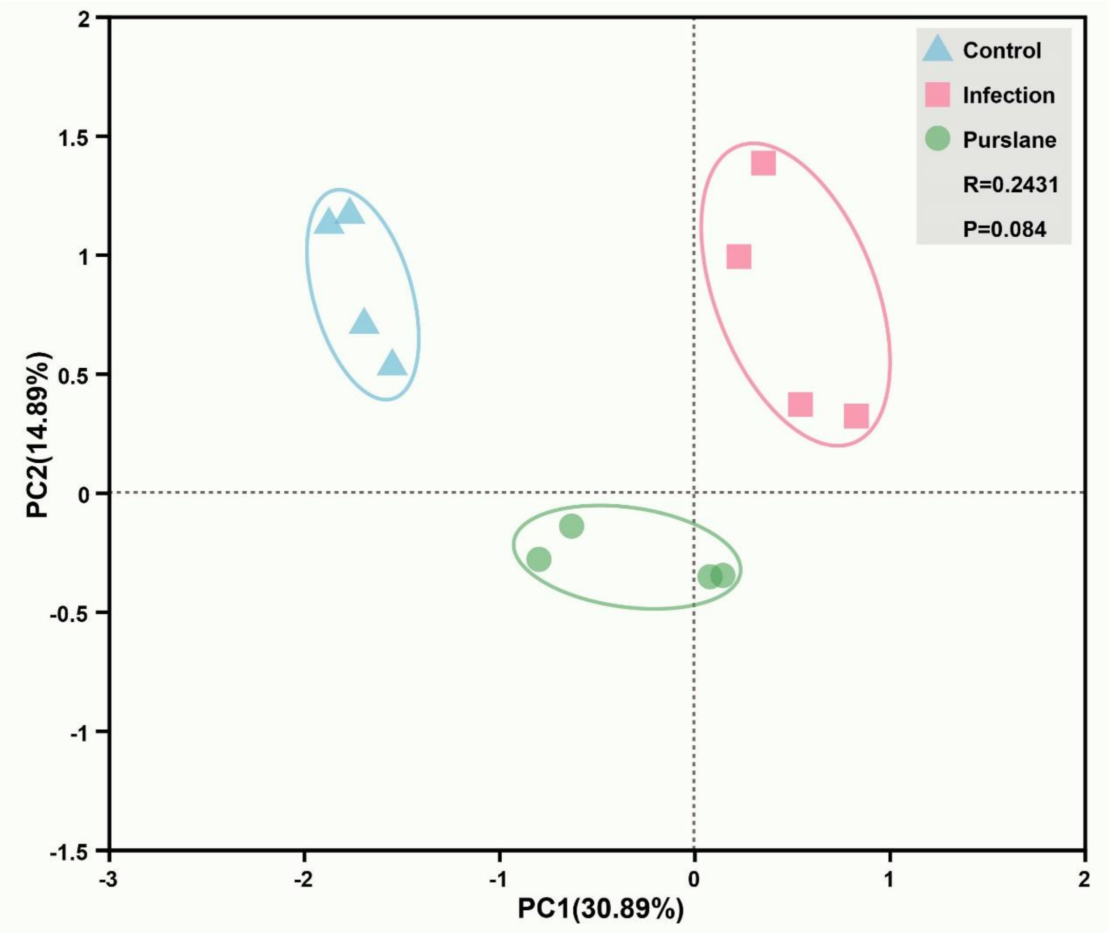

Figure S1. Principal component analysis (PCA) of samples. The OTUs of the samples were used for analysis using the Bray-Curtis algorithm.
